# Seroprevalence and risk assessment of *Toxoplasma gondii* infection in sheep and goats in North and Beqaa governorates of Lebanon

**DOI:** 10.14202/vetworld.2022.2180-2185

**Published:** 2022-09-13

**Authors:** Sara Khalife, Sara Moubayed, Rosy Mitri, Regina Geitani, Dima El Safadi

**Affiliations:** 1 Department of Medical Laboratory Technology, Faculty of Health Sciences, Beirut Arab University, Tripoli, Lebanon; 2 Department of Nutrition and Dietetics, Faculty of Health Sciences, Beirut Arab University, Tripoli, Lebanon; 3 Laboratoire des Agents Pathogènes, School of Pharmacy, Saint Joseph University of Beirut, Beirut, Lebanon; 4 Laboratory of Microbiologie Santé et Environnement, Doctoral School of Sciences and Technology, Faculty of Public Health, Lebanese University, Tripoli, Lebanon

**Keywords:** goats, Lebanon, risk factors, seroprevalence, sheep, *Toxoplasma gondii*

## Abstract

**Background and Aim:**

Toxoplasmosis is a disease caused by the protozoan parasite *Toxoplasma gondii* that affects both humans and animals, leading to abortions and significant clinical manifestations in pregnant and immunocompromised hosts, in addition to massive economic losses in animal industries. Data from Lebanon are scarce regarding the seroprevalence of *T. gondii* infection in livestock. This study aimed to estimate the seroprevalence and assess the associated risk factors of *T. gondii* infection in sheep and goats in Lebanon.

**Materials and Methods:**

A cross-sectional study was carried out from May 2020 to April 2021. Blood samples from 150 sheep and 145 goats (total 295) destined for human consumption were obtained from 20 Lebanese farms located in the North and Beqaa governorates. The anti-*T. gondii* immunoglobulin G antibodies were assayed through means of a modified agglutination test with a cutoff titer of 20.

**Results:**

An overall seroprevalence of 48.5% (143/295) was reported: About 56.6% seroprevalence was found in sheep (85/150) and 40% (58/145) in goats. Adult age, female gender, and the wet season were significantly associated with an increased seropositivity rate of *T. gondii* infection (p < 0.001, p = 0.001, and p = 0.043, respectively).

**Conclusion:**

These results confirm the spread of *T. gondii* in sheep and goats destined for human consumption in various geographical regions in Lebanon. Therefore, continuous monitoring of *T. gondii* infection in livestock is warranted to control the spread of the infection and limit its potential transmission to humans through the consumption of raw or undercooked meat.

## Introduction

*Toxoplasma gondii*, an intracellular Apicomplexan protozoan, is the causative agent of a common parasitic zoonotic disease, toxoplasmosis. Toxoplasmosis affects, as intermediate hosts, warm-blooded animals worldwide, including livestock [1, 2]. Members of the family *Felidae*, which includes domestic cats, serve as definitive hosts of *T. gondii* [3]. Toxoplasmosis is transmitted to humans through the consumption of raw or undercooked meat infected with bradyzoites in intracellular tissue cysts, or by the ingestion of sporulated oocysts shed by infected definitive hosts in soil, water, fruits, and vegetables [4].

The highest prevalence of *T. gondii* infection among meat-producing animals is reported in sheep and goats [5]. Therefore, the ingestion of infected uncooked milk or raw or undercooked meat from these animals could be potentially hazardous for immunocompromised individuals, including pregnant women. Indeed, pregnant women with primary *Toxoplasma* infection could suffer from pregnancy loss or severe manifestations in the newborn following transplacental transmission [4]. Moreover, *T. gondii* infections are an important cause of neonatal mortality in sheep and goats, resulting in economic burdens worldwide.

Pregnant Lebanese women have the highest seroprevalence of anti-*T. gondii* immunoglobulin G (IgG) in the Arab region, reaching 82.6%, with a rate of primary *Toxoplasma* infection during pregnancy estimated at 1.8% [6]. The prevalence of *T. gondii* in meat-producing animals has been widely reported across the world [4, 7–14]. However, similar national data is limited and consists of one report examining the seroprevalence of *T. gondii* in a limited number of specimens in one geographical area in Lebanon [15].

This study aimed to estimate the seroprevalence of anti-*T. gondii* antibodies in two main livestock-production areas in Lebanon, and to evaluate the risk factors of *T. gondii* infection in sheep and goats.

## Materials and Methods

### Ethical approval and informed consent

The ethical approval was waived because the study used routine procedures to collect the diagnostic data. The authors declare that they have followed the European standards (Directive 2010/63/EU) on the protection of animals used for scientific purposes. All the farm owners completed the consent form to include their animals in this study.

### Study period and location

The study was conducted from May 2020 to April 2021. This cross-sectional study was conducted in the North and Beqaa governorates of Lebanon.

### Study design and samples

A total of 295 animals were included in this work, comprising 150 sheep and 145 goats. Blood samples were obtained from the jugular veins of selected animals. Sera were prepared by centrifugation at 2000× *g* for 10 min, and stored at –20°C until serological testing.

Data, including species, age, gender, management practices, geographical area, season and presence of cats in the farm or grazing areas, were obtained for a risk factor analysis of *T. gondii* infection. Animals aged <1 year were considered young, while those above 1 year were considered adult.

### Study animals

The research was conducted on 150 sheep and 145 goats. The study animals originated from 20 different farms located in two governorates of Lebanon: 145 animals were randomly selected from ten farms in North Lebanon governorate, and 150 animals were arbitrarily chosen from ten farms located in the Beqaa governorate of Lebanon. Eighty-eight young sheep and goats of different breeds versus 207 adults were included in this study. One hundred and ten males were involved versus 185 females. Semi-intensive husbandry system and animals’ grazing were frequent management practices reported in the tested farms.

Regarding the season, 125 versus 170 animals were sampled in the wet and dry seasons, respectively (Table-1).

**Table-1:** Seroprevalence and associated risk factors of *T. gondii* infection in sheep and goats in Lebanon.

Variables	Category	No. examined	No. positive	Seroprevalence (%)	OR (95% CI)	p-value
Species	Sheep	150	85	56.6	1.961 (1.234–3.117)	0.004^†^
	Goats	145	58	40		
Age (years)	Young	88	25	28.4	3.341 (1.949–5.726)	<0.001^†^
	Adult	207	118	57		
Gender	Male	110	40	36.4	2.198 (1.353–3.569)	0.001^†^
	Female	185	103	55.7		
Geographical area	North	145	76	52.4	1.364 (0.863–2.157)	0.183
	Beqaa	150	67	44.6		
Seasons	Dry	125	52	41.6	1.617 (1.014–2.577)	0.043^†^
	Wet	170	91	53.5		
Feeding type	Concentrates/Hay	55	27	49.09	1.030 (0.573–1.852)	0.919
	Grazing	240	116	48.33		
Farming management	Extensive	70	39	55.71	1.269 (0.740–2.176)	0.385
	Semi-intensive	225	112	49.77		

† Statistically significant, *T. gondii=Toxoplasma gondii*, CI=Confidence interval, OR=Odds ratio

### Serological testing

The serological testing has been carried out at the Biomedical Sciences Laboratory of Beirut Arab University. The IgG antibodies specific to *T. gondii* were determined by a modified agglutination test (MAT), using a commercial kit (Toxo-Screen DA®, bioMerieux, Lyon, France) according to the manufacturer’s protocol, as described previously [16].

Two-fold serial dilutions were performed from 1:20 to 1:2560. Positive and negative controls supplied by the kit were included in each testing plate. A cutoff titer of 1:20 was set to improve both the sensitivity and specificity of the test.

### Statistical analysis

All statistical analyses were performed using IBM Statistical Packages for the Social Sciences for Windows, version 22 (IBM SPSS, IBM Corp., Armonk, NY, USA). The Chi-square or Fisher exact test was used to assess the association of the *Toxoplasma* seroprevalence with specific risk factors. p < 0.05 was considered to be statistically significant.

## Results

One hundred and forty-three animals out of the 295 tested showed seropositivity to *T. gondii*, leading to an overall seroprevalence of 48.5% (143/295) (95% confidence interval [CI]: 0.426–0.543). Positive samples yielded positive results for both dilutions tested (1:20 and 1:2560).

As shown in Table-1, the seroprevalence of *T. gondii* was significantly higher in sheep – 56.6% (85/150) – than in goats – 40% (58/145) (odds ratio [OR] = 1.961; 95% CI: 1.234–3.117) (p = 0.004).

A statistically significant higher seroprevalence rate was observed in adult animals – 57% (118/207) – than in young animals – 28.4% (25/88) (OR = 3.341; 95% CI: 1.949–5.726) (p < 0.001).

Similarly, the seroprevalence was significantly higher in females – 55.7% (103/185) – than in males – 36.4% (40/110) (OR = 2.198; 95% CI: 1.353–3.569) (p = 0.001) (Table-1).

The seroprevalence of *T. gondii* infection was significantly higher in the wet season – 53.5% (91/170) – in comparison to the dry season – 41.6% (52/125) (OR = 1.617; 95% CI: 1.014–2.577) (p = 0.043).

These results could further indicate that ruminant species, age, gender, and seasonality might affect *T. gondii* seropositivity in Lebanon.

In contrast, as demonstrated in Table-1, the difference between the seroprevalence rates found in the North Lebanon – 52.4% (76/145) – and Beqaa areas – 44.6% (67/150) – was statistically insignificant (OR = 1.364; 95% CI: 0.863–2.157) (p = 0.183), suggesting that geographical regions may not be a potential risk factor of *T. gondii* infection in Lebanon.

Likewise, there was no significant difference in the seroprevalence of IgG anti - *T. gondii* antibodies according to the feeding type, or farming management ([OR = 1.030; 95% CI: 0.573–1.852] [p = 0.919]; [OR = 1.269; 95% CI: 0.740–2.176] [p = 0.385], respectively). These findings suggest that the feeding and the farming management types could not be considered as risk factors of *T. gondii* infection in Lebanon.

## Discussion

Toxoplasmosis is considered a globally prevalent parasitic disease, with a seropositivity rate ranging from <10% to over 90% in the human population [17]. It is estimated that around 22% of *T. gondii* infections in humans originated from meat-producing animals, particularly sheep, and goats [18], with a high infection rate reaching up to 92% and 75%, respectively [19]. Thus, screening for such zoonosis is highly recommended to halt the transmission of *T. gondii* to humans [20]. The present study has, therefore, examined the seroprevalence of *T. gondii* infection in the small ruminant’s population in Lebanon, in both North and Beqaa governorates, and evaluated the risk factors associated with this infection.

The overall seroprevalence of *T. gondii* in livestock obtained in this study, accounting for around 48.5% (143/295), is comparative to the figure of 42.2% (76/180) obtained in a previous study conducted in the North Lebanon governorate [15]. In addition, our results are consistent with other reports in Pakistan and Spain, where the seropositivity rates of *T. gondii* in the examined ruminants were 42.80% (107/250) and 42.5% (826/1943), respectively [21, 22]. Moreover, a study conducted in Egypt has shown a similar seroprevalence of *T. gondii* in sheep and goats, with infectivity levels of 46.6% (97/208) and 52.4% (109/208), respectively [23].

However, other studies have reported lower *T. gondii* seropositivity in comparison to our findings. For example, an overall seropositivity rate of 11.6% (21/180) was reported in slaughtered sheep and goats in Central Iran [24]. Similar findings were reported in Italy, Columbia, Turkey, and Bangladesh, indicating remarkably low seropositivity to *T. gondii* antibodies in meat-producing animals, reaching 28.4% (79/278), 23.5% (244/1038), 18% (154/859), and 13% (114/852), respectively [25–27].

On the other hand, a study conducted in Northwest Ethiopia reported a higher IgG anti-*T. gondii*, at a level of 70.48% (406/576), in the sera of small sheep and goats, compared with our findings [28].

The discrepancy in the reported seroprevalence of *T. gondii* in small ruminants in different countries could be attributable to various factors, including the sampling strategy, which generally affects the representativity and accuracy of the data; the management system; the level of animals’ access to contaminated water and feeding sources; geo-climatic variations; hygiene and precautionary measure; the frequency of the cat population in the area surrounding the farms; the serological testing techniques and cutoff values used; and the adopted protocol for data analysis. In this study, the relatively high *T. gondii* seropositivity in tested animals in the North and Beqaa governorates of Lebanon could be associated mostly with environmental and hygiene conditions. The mild climatic conditions in Lebanon, in terms of humidity and temperature, promote the spread, transmission, and infection cycle of *T. gondii* by affecting the survival rate of parasitic oocysts. Moreover, all the involved farms were characterized by an open household system, which facilitates direct contact with cats and their eventual excreted oocysts, which may contaminate the soil, water, and feeding sources of small ruminants.

In the present study, the MAT assay was used as a screening method for the detection of anti-*T. gondii* IgG has a higher sensitivity and specificity than the other serological techniques used for the serodiagnosis of *T. gondii* infection, including the Latex Agglutination test, the dye test, the indirect immunofluorescence test, and the enzyme-linked immunosorbent assay (ELISA). In fact, a comparative study conducted in Egypt has reported a higher sensitivity (96%) and specificity (88.9%) rate using the MAT assay for the detection of toxoplasmosis in a total of 300 slaughtered sheep, as compared to the ELISA technique, which elicited sensitivity and specificity levels of 90.1% and 85.9%, respectively [29].

In addition, this study has indicated that sheep carried higher anti-*T. gondii* IgG in sera compared to goats, with seropositivity levels of 56.6% (58/150) and 40% (58/145), respectively. These findings are in accordance with a study conducted in Ghana, where sheep were found to be more susceptible to *T. gondii* infection, with a seropositivity level of 35.9% versus 23.7% in goats [30]. Similarly, a recent report in Ethiopia demonstrated higher seropositivity for anti-*T. gondii* IgG in sheep (76%) than in goats (65%) [28]. This was in line with several other studies across the globe [13, 30–33]. It could be explained by the grazing pattern of sheep, favoring contact with the sporulated oocysts and increasing the risk of parasitic infection, as compared to goats, which have a preference for browsing. In addition, the co-domestication of household pets, such as cats, with the sheep could explain the high seropositivity of zoonotic infection among these ruminants. In contrast, a study conducted in Pakistan reported a higher prevalence of *T. gondii* in goats (42.8%) compared to sheep (26.2%) [34]. Similar findings were also reported in Egypt and São Tomé [14, 19]. These were mainly attributed to the husbandry strategy and herding practices of goats since they are allowed greater movement, enhancing their contact with sporulated oocysts sources, whereas sheep are usually house-kept and less exposed to parasitic contamination [34].

However, other studies conducted in Italy, Mongolia, Northern Iraq, Iran, and Pakistan did not support the association between *T. gondii* seropositivity and animal species [13, 22, 25, 35, 36]. This was mainly due to the fact that all livestock were herded together on open grassland with river water [13].

Furthermore, our study has shown a significant association between the age of the ruminants and the seropositivity to *T. gondii* antibodies, suggesting that adult animals (>1 year, 57%) are considered at a higher risk of acquiring *T. gondii* infection in comparison to the younger group (≤1 year, 28.4%). This was in accordance with a study conducted in Mongolia, where young goats of 1–2 years old were found to have a higher *T. gondii* seroprevalence (39.7%) compared to older animals (3–4 years old, 27.9%; 5–6 years old, 28.3%) [13]. Other studies conducted in Algeria, Pakistan, São Tomé, Bangladesh, and South Africa have demonstrated similar results [14, 25, 27, 28, 32, 37, 38]. This variation could be due to the repetitive exposure of adult animals to oocysts from the environment for a longer period of time than younger animals. However, several studies conducted in West Africa, Iran, Egypt, and Pakistan did not find an association between the age of the sheep and goats and the incidence of toxoplasmosis [22, 24, 31].

In this study, females were found to be more susceptible to *T. gondii* infection in comparison to males (55.7 vs. 36.4%, respectively) in both sheep and goats. This finding is supported by several other studies [22, 34, 35], including a recent report conducted in Southern Ethiopia, where gender was considered a potential risk factor for the acquisition of *T. gondii* infection in sheep ruminants, with a higher seropositivity level reported in females than males of 67.2% versus 43.6%, respectively [39]. The higher incidence of toxoplasmosis in the ewe population is generally due to pregnancy-associated immunosuppression, lactation stress, physiological conditions, and hormonal fluctuations. However, contradictory findings have also been reported elsewhere [13, 24, 26, 31].

In our study, the geographical location did not seem to affect *T. gondii* seropositivity rate. This was in agreement with other studies conducted in Pakistan and Iraq [22, 35]. However, other data have shown that geographical location could be an important risk factor for toxoplasmosis in the sheep and goat populations [40].

In our study, seasonality seems to affect *T. gondii* seropositivity rate. Indeed, a significantly higher seropositivity rate was reported in the wet season as compared to the dry season: 53.5% versus 41.6%, respectively. Our results are in line with the previous data reported in Iran, where *T. gondii* seropositivity among the sheep was shown to be significantly higher in wet and mild climates, which are considered the optimum conditions for the growth and survival of *T. gondii* oocysts [36]. In contrast, a study conducted in Egypt could not find a significant association between *T. gondii* seropositivity rate and climatic conditions [31].

## Conclusion

High seroprevalence of antibodies specific to *T. gondii* has been reported in sheep and goats in two main livestock production areas in Lebanon, suggesting a wide spread of oocysts in the environment, with a potential risk of transmission to humans through the consumption of raw or undercooked meats. In addition, ruminant species, age, gender, and seasonality seem to affect *T. gondii* seropositivity rates in Lebanon. However, our data cannot provide a definitive diagnosis of the infection status of the serologically positive animals. Further molecular investigations and bioassays are warranted to assess the infectivity and the potential risk of transmission of *T. gondii* to humans.

## Authors’ Contributions

SK and RG: Conceptualization, data curation, and methodology. SK, SM, RM, RG, and DE: Investigation, visualization, and writing - review and editing. SK, and SM: Writing - original draft. All authors have read and approved the final manuscript.
